# Mr. Good's System of Physiological Nosology

**Published:** 1817-11

**Authors:** 


### *Mr. Good's System of Physiological Nosology.*

( Concluded from page 301. )

### Series of Classes and Orders.

#### Class I. CŒLIACA.

*Diseases of the Digestive Function.*

Order 1. Enterica—Affecting the alimentary canal.
2. Splanchnica--Affecting the collatitious viscera.

#### Class II. PNEUMATICA.

*Diseases of the Respiratory Function.*

Order 1. Phonica—Affecting the vocal avenues.
2. Pneumonica—Affecting the lungs, their membranes, or motive power.

#### Class III. HÆMATICA.

*Diseases of the Sanguineous Function.*

Order 1. Pyrectica—Fevers.
2. Phlogotica—Inflammations.
3. Exanthematica—Eruptive fevers.
4. Dysthetica—Cachexies.

#### Class IV. NEUROTICA.

*Diseases of the Nervous Function.*

Order 1. Phrenica—Affecting the intellect.
2. Æsthetica—Affecting the sensation.
3. Cinetica—Affecting the muscles.
4. Systatica—Affecting several, or all the sensorial powers simultaneously.

CLASS V. GENETICA.

*Diseases of the Sexual Function.*

ORDER 1. Cenotica—Affecting the fluids.
2. Orgastica—Affecting the orgasm.
3. Carpotica—Affecting the impregnation.

CLASS VI. ECCRITICA.

*Diseases of the Excernent Function.*

ORDER 1. Mesotica—Affecting the parenchyma.
2. Catotica—Affecting internal surfaces.
3. Acrotica—Affecting the external surface.

CLASS VII. TYCHICA.

*Fortuitous Lesions, or Deformities.*

ORDER 1. Apalotica—Affecting the soft parts.
2. Stereotica—Affecting the hard parts.
3. Morphica—Monstrosities of birth.

Such is the general outline of Mr. Good's *plan;* and we shall now proceed to lay before the reader a suite of specimens from the *execution.*

*Order* 1. ENTERICA. Affecting the alimentary canal. Disquiet, or diseased action in some part of the passage for the reception and detrition of food. *Genus I.* ODONTIA, pain or derangement of the teeth or their sockets. Spec. 1. DENTITIONIS, irritation from cutting the teeth. [Odontiasis, *Paul. Ægin.* Odontalgia dentitionis, *Sauv.* Odaxismus, *Vog.* Ziras, *Arab.* Zahnen, *Germ.* Dentition, *Fr.*] *Teething.* α. Lactentium, cutting the milk or shedding teeth. β. Puerilis, cutting the second set, or permanent teeth. γ. Adultorum, cutting the adult or wise teeth. δ. Senilium, cutting teeth in advanced life, or old age. Sp. 2. DOLOROSA, acute pain in the teeth, or their sockets. [We now omit the *synonims* and also the *varietes,* as the above specimens are sufficient.] 3. STUPORIS, tingling pain in the teeth from stridulous sounds, vellication, or acrid substances. 4. DEFORMIS, deformity of the teeth from error of shape, position, or number. 5. EDENTULA, loss or want of teeth. 6. INCRUSTANS, teeth incrusted with extraneous matter. 7. EXCRSECENS, the substance of the surrounding gums excrescent.

*Genus II.* Ptyalismus. Involuntary flow of saliva from the mouth. Sp. 1. ACUTUS, increased secretion of saliva from increased action of the salivary glands. 2. CHRONICUS, increased secretion of saliva from debilitated habit; and relaxation of the salivary glands. 3. Iners, involuntary flow of saliva from a sluggishness of deglutition without increased secretion.

*Genus III.* Dysphagia. Pain or obstruction in swallowing without inflammation, and mostly without impeded respiration. Sp. 1. Constricta, difficulty of swallowing from permanent constriction of the œsophagus. 2. Atonica, difficulty of swallowing from debility of the muscles of deglutition. 3. Globosa, difficulty of swallowing from wind in the stomach spasmodically into the feeling of a ball, ascending into the œsophagus, and producing a sense of strangulation. 4. Uvulosa, swallowing obstructed or troublesome from relaxation and enlargement of the uvula. 5. Linguosa, swallowing obstructed or troublesome from protrusion or magnitude of the tongue.

*Genus IV.* Dipsosis. The desire for drinking excessive or impaired. Sp. 1. Avens, constant desire of drinking; with a sense of dryness in the mouth and throat. 2. Expers, constant want of thirst.

*Genus V.* Limosis. The appetite for food impaired, excessive, or depraved. Sp. 1. Avens, insatiable craving for food. 2. Expers, loss or want of appetite without any other apparent affection of the stomach. 3. Pica, appetite for improper and indigestible substances. 4. Cardialgia, impaired appetite, with gnawing or burning pain in the stomach or epigastrium, and tendency to faint. 5. Flatus, impaired appetite, with accumulation of wind in the stomach or intestinal canal, and frequent regurgitation. 6. Emesis, rejection of the contents of the stomach, or tendency to reject. 7. Dyspepsia, the appetite fastidious, and the food digested with difficulty.

*Genus VI.* Colica. Griping pain in the bowels, chiefly about the navel, with vomiting and costiveness. Sp. 1. Ileus, with retraction of the navel, and spasms of the muscles of the belly. 2. Rachialgia, the pain at first dull and remitting; but progressively growing more violent and continued; extending to the back and arms; and at last producing paralysis. 3. Crapulosa, the pain accompanied with nausea, head-ache, and dizziness, before vomiting, and often terminating in a griping looseness. 4. Flatulenta, the pain acute; extending to the pit of the stomach, often impeding respiration; accompanied with great fulness and flatulency; and relieved by pressure, bending the body forward, or expulsion of wind. 5. Stipata. The pain severe; the costiveness obstinate; great tension, with little flatulency: the vomiting sometimes accompanied with fæces; the costiveness with bloody strainings: terminating, where not fatal, in a free dejection of the infarcted matter. 6. Callosa, with a sense of stricture in some part of the intestinal canal; often of flatulency and pain, the flatulency gradually passing off by the stricture; the bowels tardy; at length discharging small liquid stools.

*Genus VII.* Coprostasis. Obstinate retention of the fæces in the intestines. Sp. 1. Coacta, the fæces, when discharged, impacted and voluminous; the temperament firm and rigid. 2. Adstricta, the fæces, when discharged, hard, slender, and often scybalous; the temperament weakly, or the habit sedentary.

*Genus VIII.* Dysenteria. Griping and tenesmus; frequent mucous, and often bloody dejections; the fæces seldom discharged, and in small quantities. Sp. 1. Simplex, unaccompanied with fever: the fæces, when discharged, evacuated without considerable pain, of a natural quality, and affording ease. 2. Pyrectica, accompanied with fever, great loss of strength, and depression of spirits: the fæces, when discharged, of various colours and consistence; highly fœtid, and mixed with putrid sanies, seba ceous matter, or membranous films.

*Genus IX.* Diarrhœa. Alvine evacuations crude, loose, and too frequent; with little or no griping or tenesmus. Sp. 1. Fusa, fæces of common quality, but immoderately loose, and copious. 2. Biliosa, fæces loose, copious, and peculiarly yellow. 3. Mucosa, the dejections consisting of, or containing a copious discharge of mucus. 4. Chylosa, the dejections milky or chyliform. 5. Lienteria, the dejections consisting of the aliment passed rapidly and with little change. 6. Serosa, the dejections almost entirely liquid.

*Genus X.* Cholera. Anxiety, gripings, spasms in the legs and arms; for the most part with bilious vomiting and purging. Sp. 1. Vulgaris, the vomiting and purging frequent and copious. 2. Flatulenta, the vomiting and purging, rare or absent; great and oppresive flatulence; retching; flatulent dejections and eructations. 3. Spasmodica, the dejections watery; ineffectual retching; spasms successive and violent, commencing in the thoracic and abdominal muscles.

*Genus XI.* Enterolithus. Stony concretions in the stomach or intestinal canal. Sp. 1. BEZOARDUS, in concentric layers closely agglutinated or crystallized; capable of a fine polish, frequently with a metallic lustre on the surface of each layer; and an accidental nucleus in the centre; of a spheroidal figure; chiefly consisting of vegetable matter. 2. CALCULUS, radiating from a common centre, or formed in concentric layers; mostly with an accidental nucleus; more or less porous; spheroidal or oblong, admitting an imperfect polish; composed chiefly of earths and animal matter. 3. SCYBALUM, soapy or unctuous; mostly continous; sometimes in layers; spheroidal or oblong; varying in colour; consisting chiefly of mucous and oleaginous matter.

*Genus XII.* Helminthia. Worms or larves of insects inhabiting the stomach or intestines. Sp. 1. ALVI, worms existing and finding a proper nidus in the stomach or alvine canal, chiefly of children and sickly adults; producing emaciation, a swelled hard belly, gnawing or pungent pain in the stomach, pale countenance, fœtid breath, and irritation of the nostrils. 2. PODICIS, worms, or the larves of insects existing, and finding a proper nidus within the verge of the anus, exciting a troublesome local irritation, sometimes accompanied with tumour; frequently preventing sleep, and producing pain or faintness in the stomach. 3. ERRATICA, worms introduced by accident, and without finding a proper habitation in the stomach or intestines: producing spasmodic colic with severe gripings; and occasionally vomiting or dejection of blood.

*Genus XIII.* Proctica. Pain or derangement about the anus, without primary inflammation. Sp. 1. SIMPLEX, simple pain at the anus. 2. CALLOSA, pain produced by, or accompanied with, a callous contraction of the rectum. 3. TENESMUS, painful and perpetual urgency to go to stool, with dejection of mucus alone, and in small quantity. 4. MARISCA, livid and painful tubercles or excrescences, usually with a discharge of mucus or blood. 5. EXANIA, inversion and prolapse of the villous tunic of the rectum, from relaxation of the sphincter, with more or less tumour.

The foregoing ORDER will prove a sufficient specimen of the *Nosological Arrangement* of Mr. Good's work; but we are sorry that our limits and the nature of a Medical Journal, will not permit us to introduce samples of the learning and research of the able author. To the work itself we must, therefore, refer on these, as well as on several other points. The remainder of this Article shall be dedicated to selections from the immense body of Notes, which indeed forms the most valuable, and, in our eyes, attractive feature of this masterly performance.

1. *Ptyalismus.* [*page* 11.] As a critical discharge, salivation is, for the most part, salutary, and often terminates the disease that excites it. This is frequently the case in fevers, and the following instance is perhaps worth relating. A lady, aged 24, and of a delicate constitution, was attacked with a typhus in the spring of 1788, under which she gradually drooped for nearly three weeks. The author thought her in great danger; but on the twentieth day, a sudden and copious ptyalism supervened, that evidently afforded her considerable relief. This continued for upwards of a week, the daily secretion being never less than a pint, and twice not less than a pint and a quarter. Yet, instead of adding to her debility, it appeared to give fresh vigour to the system: the digestive function resumed its office; she daily improved in strength; and on its cessation at the above period, was in a state of convalescence.

The secretion of sweet or mawkish saliva is not only for the most part free, but accompanied with nausea, and other symptoms of indigestion: and is probably what Sauvages intends for his first species, *p. nauseosus,* or *a saburrâ nidorosâ.* It is relieved by magnesia and other absorbents; but will often only yield to an emetic, followed by warm stomachics. The author has not found acids of more than palliative service, and has sometimes thought the complaint worse from their exhibition. p. 13.

2. *Nausea and Vomitus, (page* 27.*)* It is, nevertheless, curious and of great importance, to observe the different and opposite effects produced on the animal frame by these two stages of one and the same disease. Nausea lowers the pulse, contracts the small vessels, occasions cold perspiration, severe rigors and trembling, and diminishes, as long as it lasts, the action and even the general powers of life. The act of vomiting, on the contrary, rouses rather than depresses; puts to flight all the preceding symptoms, and restores the system to itself. There are few persons so debilitated as not to bear vomiting, but many who would soon sink under nausea. It is obvious, therefore, that these two different states of the stomach may be employed as powerful instruments in attacking a variety of general and even of remote local diseases, this organ being justly considered as the common centre of sympathy, and producing opposite results, according as it is excited to different degrees of action.

3. *Colica Callosa, (page* 39.*)* The stricture here referred to is the *scirrho-contracted rectum* of surgical writers. It lies beyond the reach of topical remedies, and is chiefly to be alleviated by a rigid attention to light, liquid, and aperient diets. The disease is a lamentable, though not a common one; yet the writer of this work has, at the present time, three patients afflicted with it under his care; one, a lady of twenty-four years of age, who has been a sufferer for about two years; another, a lady of thirty-five, who has been subject to it for ten years; neither of whom is capable of passing fæces more voluminous than those of an infant; and the third a man of forty-nine years old, who has laboured under the disease for twenty-one years, and can never pass a motion larger than a crow-quill. Yet, by strict attention to diet, all three are able to exist with only occasional inconvenience and pain; the last married about two years since, and his wife has lately brought him twins. He lives upon liquids altogether.

4. *Coprostasis Adstricta, (page* 40.*)* In the case of a young woman aged twenty-eight, the distention of the abdomen was so general as to be mistaken for pregnancy, especially as there was occasional sickness, menstrual suppression, and sympathetic enlargement of the breasts. The case terminated fatally in about three years from its commencement. The colon, preserved by Mr. Taunton, who has obligingly shewn it to the author, as well as favoured him with this history, measures in circumference more then twenty inches, and on dissection was found to contain three gallons of fæces.

5. *Diarrhœa Serosa, (page* 46.*)* A young woman, aged twenty-four, applied to the author in August 1806, with a disorder of this species, which had continued ten years, and never produced fewer than nine or ten watery stools a-day, sometimes tinged with blood. She was often in great spasmodic pain in the stomach or intestines; and had tried a long list of astringents, anodynes, and other medicines, to little purpose. She was much reduced, and it appeared to be a case of great local irritation from local debility. Gentle stimulants were here of essential service, and the disease gradually yielded to camphor mixture and pills of the resinous gums.

" 6. *Lyssa, (page 352.)* Λύσσα, " rabies" whence λυσσω, " fuio;" probably from λύω, " solvo." The old Greek term is here restored, as far more correct than that of the present day, which is *hydrophobia* or *water-dread*, since the last is not a pathognomic symptom, being sometimes found in other diseases; occasionally ceasing even in canine madness, the second species here offered, towards the close of the paroxysm; and though almost always found amongst mankind, in numerous instances wanting in rabid dogs and wolves. "Constat repetitâ," says Sauvages, "apud Gallo-provinciales experientiâ, canes luposque rabidos bibisse, manducasse, flumen tranasse, ut olim Marologii et bis Forolivii observatum, adeoque nec cibum nec potum aversari." According to Meynell, the disease among dogs appears from ten days to eight months after the bite. In Earl Fitzwilliam's hounds, which were bitten June 8, 1791, the interval varied from six weeks to more than six months. Among mankind its accession is also very uncertain, as this seems to be regulated by the time of the year, the habit of the patient, and other accidental circumstances. It has occurred a fortnight after the bite, three weeks, a month, and sometimes six weeks, and even three months; after which last period the patient is considered safe.

"In two cases published by Dr. Tracher in the *American Medical and Philosophical Register*, Vol. I. page 457, the injury inflicted by the same dog, August 16th, 1810, did not produce hydrophobia till nearly three months afterwards, November 3d, and November 14th: the first case was that of a child under four years of age, the second that of an old man of seventy-three. In the former the hydrophobia continued for six days, and in the latter for seven, before death ensued. The remedies chiefly recommended in America are the lobelia *inflata*, scutellaria *latiflora*, anagallis *arvensis*, in the form of tincture. Dr. Hossack frankly confesses he has no confidence in any of them.

"In the Memoirs of the Manchester Society, Vol. 4, is a singular case published by Dr. Bardesley, of an attack of lyssa twelve years after the bite of a dog supposed to be mad. The patient died in the Manchester Infirmary with the most decided symptoms of the disease. From the long interval between the bite and the symptoms, Dr. Bardsley is more inclined to ascribe the disease to several existing circumstances at the time of its appearance, and particularly to a deep depression of spirits and strong mental agitation the patient had for some time been labouring under, than to any canine poison which might be lurking in the constitution. But this would be to suppose that lyssa is capable, under particular circumstances, of being spontaneously generated even in the human frame, while Dr. Bardsley contends that it cannot exist even *among dogs*, except by contact."

How far Mr. Good may be successful in persuading the medical world to adopt his *terms* as well as his arrangements, we do not pretend to know But as grey hairs do not relish the trouble of going to school again, it is probable, that our author must trust principally to the rising generation for encouragement in this respect. In fine, we consider Mr. Good's nosological arrangement of diseases as better adapted for the *memory,* than any preceding system with which we are acquainted; and as for his *terminology,* it is infinitely superior in euphony, derivation, and classical purity, to any thing of the kind, in the annals of English medical literature.

